# Reduced Cyclic Adenosine Monophosphate Level in Hippocampal CA1 Participates in Propofol Induced Amnesia in Rats

**DOI:** 10.3389/fnins.2018.00337

**Published:** 2018-05-23

**Authors:** Weiwei Li, Lingling Yu, Xiaodi Yan, Linlin Cai, Li Wan, Qinyu Teng, Yonghua Li, Yun Wang, Haitao Xu

**Affiliations:** ^1^Department of Anesthesiology, Changzheng Hospital, Second Military Medical University, Shanghai, China; ^2^Institutes of Brain Science & State Key Laboratory of Medical Neurobiology, Zhongshan Hospital, Fudan University, Shanghai, China; ^3^Department of Anesthesiology, Xinhua Hospital, Shanghai Jiaotong University, Shanghai, China; ^4^Department of Critical Care Medicine, Zhongshan Hospital, Fudan University, Shanghai, China

**Keywords:** propofol, amnesia, cyclic adenosine monophosphate, phosphodiesterases 4, hippocampus

## Abstract

Propofol inhibits long-term potentiation (LTP) in the hippocampal CA1 region and impedes episodic memory formation. However, the molecular mechanisms involved in the effect of propofol are still poorly understood. It had been reported that propofol inhibited cAMP response element binding protein signaling, which was proposed to contribute to memory retention impairment in rats. Here, we first demonstrated that propofol perfusion could inhibit forskolin induced LTP in the rat hippocampal CA1 slices. Propofol also reduced the level of cAMP, which could be reversed by non-selective PDE inhibitor IBMX. We further discovered that propofol could increase both PDE4 activity and PDE4AX protein expressions in the hippocampal CA1 region. Furthermore, pretreatment of rolipram, a PDE4 inhibitor, rescued propofol induced inhibition of CA1 LTP and the impairment of hippocampus-dependent memory formation in rats. Thus, our results suggest that reduced levels of cAMP by increasing PDE4 activity and PDE4AX protein expressions in the hippocampal CA1 region plays an important role in the propofol-induced amnesia.

## Introduction

Propofol, the most widely used intravenous general anesthetic, can impede episodic memory formation even at sedative doses (Pang et al., 1993; Veselis et al., 2004; Zhang et al., 2013). That is to say patients could preserve conscious awareness when subanesthetic propofol is present, but have no recollection of these events after the fact. It is known that the formation of episodic memories depends on the hippocampus, a region of the brain critical to memory formation (Wixted et al., 2014; Moscovitch et al., 2016). However, the change induced by propofol in the hippocampus related to the memory formation remains unknown.

Long-term potentiation (LTP), which is the long-lasting increase in synaptic strength following trains of stimuli, has been proposed to be as a cellular mechanism of memory formation in the brain (Eichenbaum, 1996; Miller and Mayford, 1999). Previous researches from ourselves and others found that propofol could inhibit theta burst stimulation induced hippocampal CA1 LTP in both rats and mice (Nagashima et al., 2005; Takamatsu et al., 2005; Li et al., 2014), but had little effect on the CA1 LTP evoked by high frequency stimulations (Li et al., 2014). It has been proved that different protocols induced LTP have different underlying molecular mechanisms (Nguyen and Kandel, 1997; Staubli et al., 1999; Costa and Grybko, 2005; Li et al., 2014). For example, theta burst stimulation induced LTP in hippocampal CA1 region is reported cAMP signaling dependent and sensitive to be affected by GABA receptor activation (Nguyen and Kandel, 1997; Staubli et al., 1999; Costa and Grybko, 2005). On the contrary, high frequency stimulation induced LTP is cAMP signaling independent and not sensitive to being affected by GABA receptor activation. Thus one of the differences, such as cAMP signaling and effect by the GABA receptor activation between these two forms of LTP, could likely be a contributor to this differential effect of propofol on LTP as well as memory formation.

Many findings support the view that cAMP signaling pathway mediates synaptic plasticity, such as hippocampal LTP, in rodents which is responsible for learning and memory (Wong et al., 1999; Nguyen and Woo, 2003; Wang et al., 2004). cAMP levels in cells are positively regulated by adenylyl cyclases (ACs) and negatively affected by cyclic nucleotide phosphodiesterases (PDEs). An increase in cAMP level activates protein kinase A (PKA) that, in turn, phosphorylates target proteins including kinases and transcriptional factors, such as cAMP response element-binding protein (CREB) (Kandel, 2012). Our previous study had reported that propofol could suppress the CREB phosphorylation and the expression of brain-derived neurotropic factor and activity-regulated cytoskeleton-associated proteins in the hippocampus of rats (Zhang et al., 2013). Additionally, it was reported that GABA(A) receptors could modulate the effect of cAMP on synaptic transmission and therefore determine the direction of synaptic plasticity (Yu et al., 2001). These findings indicated that propofol could either directly or indirectly affect the downstream of cAMP signaling and its functions.

Based on these findings, we hypothesized that amnesia caused by propofol might through suppress cAMP signaling in the hippocampal CA1 region. In this aspect, we had performed electrophysiological, biochemical and behavioral experiments, and discovered that propofol inhibited cAMP signaling by enhancing PDE 4 activation and PDE4AX expression in the hippocampus.

## Materials and methods

### Animals and drugs

The animal experiments were approved by the Institutional Review Board for animal research and performed according to the guidelines for animal use in laboratories established by Fudan University and Second Military Medical University. All rats, purchased from SLAC laboratory animal company (Shanghai, China), were maintained on a 12 h light/dark cycle with food and water provided *ad libitum*.

PDE Activity Assay Kit (Colorimetric) was purchased from Abcam Company (Shanghai, China). Forskolin(FSK), 3-Isobutyl-1-methylxanthine(IBMX), Rolipram, Dimethyl sulfoxide (DMSO), N-(2,6 dimethylphenylcarbamoylmethy l), propofol, intralipid and cAMP Enzyme Immunoassay Kit were purchased from Sigma-Aldrich Co. LLC (Shanghai, China). Propofol was dissolved in DMSO, and the final concentration was 0.1% in the superfusing artificial cerebrospinal fluid (ACSF). RAMH and the other drugs were administered by addition to ACSF in the *in vitro* experiment. For the *in vivo* experiment, propofol (10 mg/ml) was purchased from B. Braun (Shanghai, China).

### Hippocampal slice preparation

Sprague-Dawley rats (21–28 days old) were decapitated following diethyl ether anesthesia. The brain was rapidly removed and placed in cold normal ACSF, saturated with 95% O2/5% CO_2_ mixed gas. The collected hippocampal tissue (350 um) was immediately sliced using a vibrotome (Lecia, Nussloch, Germany). Slices were recovered in an incubation chamber for 30 min at 32°C. They were placed at room temperature for at least 60 min, before being transferred to a recording chamber perfused with gassed ACSF.

### Electrophysiological recordings

The detailed protocol for recording field excitatory post-synaptic potentials (fEPSPs) had been described previously (Li et al., 2014). The brain slices were perfused with normal oxygenated ACSF, which contained (in mM): 126 NaCl, 2.5 KCl, 1.0 NaH_2_PO_4_, 26 NaHCO_3_, 2.5 CaCl_2_, 1.3 MgCl_2_, and 10.0 glucose, with the pH adjusted to 7.4. To obtain fEPSPs from the s. radiatum of the CA1 region, bipolar stimulating electrodes were placed in the Schaffer collateral pathway, and 5–8 MO glass electrodes filled with normal ACSF were placed on the s. radiatum for recording. The stimulus intensity was adjusted to evoke 40–50% of the maximum amplitude of fEPSPs. In all experiments, the baseline of synaptic transmission was recorded steadily for at least 30 min before drug administration or delivery of the stimulus. For LTP induction, we utilized FSK perfusion protocol by perfusing 50 uM FSK for 20 min, and theta-burst stimulation (TBS) protocol, which consisted of 10 bursts of four pulses at 100 Hz, applied at 5 Hz. The strength of synaptic transmission was determined by measuring the maximum slope of the fEPSPs.

### cAMP concentration assay in the CA1 region of hippocampal slices

There were three small experiments in this part and each experiment had two groups: control group (1% DSMO) and propofol group (50 uM). The slices were incubated at normal ACSF in the experiment 1. The slices were treated for 15 min with FSK (50 uM) and FSK plus IBMX (30 uM) in the experiment 2 and 3 respectively. At 15 min after FSK or FSK plus IBMX treatment, slices were transferred to chambers with cold ACSF and CA1 region was dissected. The harvest tissues were stored in liquid nitrogen immediately. Protein concentrations were determined by the BCA method according to BCA Protein Assay protocol (Pierce BCA Protein Assay Kit, Thermo Fisher, Shanghai, China). cAMP concentration assay was performed according to the instructions of cAMP Enzyme Immunoassay Kit (Sigma-Aldrich Co. LLC, Shanghai, China).

### PDE4 activity assay in rats' hippocampal CA1 region

Twenty-four rats, aged 10–12 weeks, were randomly divided into four groups: control group at 30 min (intralipod, 5 ml/kg, i.p.), propofol group at 30 min (25 mg/kg, i.p.), control group at 120 min and propofol group at 120 min. Rats were decapitated 30 min and 120 min after the propofol injection respectively. The CA1 region of hippocampal tissues was harvested in cold ACSF and homogenated in a protein extraction reagent. Protein concentrations were determined by the BCA method according to BCA Protein Assay protocol. PDE4 activity assay was according to the instructions of PDE Activity Assay Kit (Abcam, Shanghai, China).

### PDE4 protein expression in in rats' hippocampus by immunoblotting

Western blot analysis was performed as previously described (Zhang et al., 2013). Samples from the previously described preparation were loaded and separated by SDS-PAGE, and then electrophoretically transferred to Poly vinylidene fluoride (PVDF) membranes (Millipore). Loaded PVDF membranes were incubated with primary antibodies (ab14628, 1:1,000, abcam) in 5% skimmed milk-TBS-T solution (20 mM Tris, pH 7.6, 137 mM NaCl, 0.05% Tween 20) overnight at 4°C, followed by incubation with peroxidase-conjugated affinipure goat anti-rabbit (#111-035-003, 1:20,000; Jakson) secondary antibody in TBS-T buffer. Bands were visualized by using an ECL system (Pierce). The immunoreactivity of individual band was measured by Imagepro plus (IPP) were normalized to β-actin (#94725S, 1:2,000, CST).

### Contextual fear conditioning

The protocol of fear conditioning experiment is similar to the previous described (Curzon et al., 2009). Briefly, on the training of day 1, rats were give a 120 s habituation period before cue and shock trails begins. Then, a tone cue is presented at a level of 80 dB for 15 s. A foot shock (1 mA) was administered during the last 2 s of the tone presentation. After 120 second intervals, the tone and shock presented again 4 times. On the task test of day 2, rats were gently placed into the conditioning chamber for 180 s for contextual memory at the same time and then moved to an altered chamber to test non-contextual memory. After 60 min, rats were transferred to a new location for cue test. Rats were allowed to habituate for 3 min and then the same tone cue was given for 15 s. After 60 s intervals, the tones were repeated for 2 times. Computer-controlled cameras captured the behavior of rats during the experiment course. Memory function was assessed by analysis of freezing time.

### Data and statistical analysis

Field EPSPs were recorded and analyzed by the pCLAMP software system (Molecular Devices, Sunnyvale, CA, USA). The slope of the fEPSPs during the 5 min prior to induction of LTP was taken as the baseline, and all values were normalized to this baseline.

Data collected in this study were expressed as the mean ± SEM. Statistical significance was determined by a two-tailed unpaired Student's *t*-test or one-way or two-way ANOVA with a *post-hoc* Tukey test using SPSS 20.0 (IBM Corporation, Armonk, NY, USA). *P* < 0.05 was considered to be significant.

## Results

### Propofol inhibits FSK induced LTP in hippocampal CA1 region

**Figure 1:**
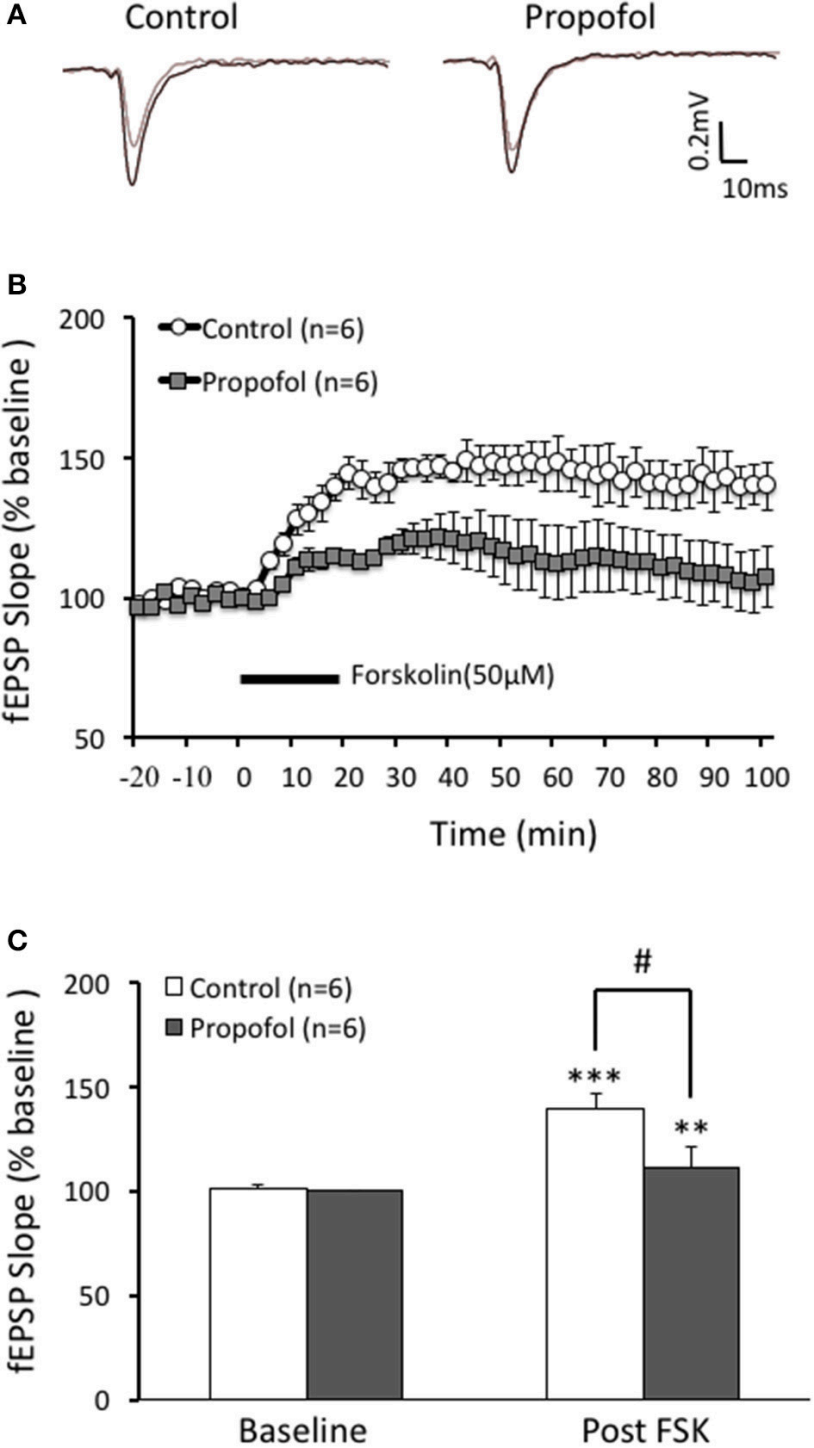
Propofol inhibits forskolin (FSK) induced LTP in the hippocampal CA1 neurons. **(A)** Traces showing of the recording of the evoked field potential 5 min before (gray line) and 80 min after FSK perfusion (black line). **(B)** Perfusion of FSK (50 μM) for 20 min sharply induced a long lasting increases of the field potential (F-LTP) in the hippocampal CA1 neurons (open circle, control group), and pretreatment with propofol (30 μM) 20 min before FSK perfusion (solid square, propofol group) significantly suppressed the FSK induced F-LTP. **(C)** Bar histogram showing group data (baseline and post-FSK) of the propofol effect on FSK induced LTP taken from 80 to 90 min after FSK perfusion. ^**^p < 0.01, ^***^p < 0.001 compared with baseline by Student's *t*-test; ^#^*p* < 0.05 compared between FSK group and FSK+Propofol group.

Our previous reports showed that treatment with propofol significantly suppressed hippocampal CA1 LTP induced by TBS, a cAMP signaling dependent LTP induction protocol, but not HFS, a cAMP signaling independent LTP induction protocol. In current study, we investigated whether the differentiation of propofol on two different protocols induced LTP was due to modulation of the cAMP levels in hippocampal neurons. FSK is an activator of the enzyme adenylyl cyclase of which activation could increase intracellular cAMP levels. FSK perfusion (50 uM) for 20 min induced a long lasting and significant increase of the fEPSP in the CA1 region of the hippocampus. The slope of fEPSP increased to 139 ± 8% of baseline (*n* = 6, *p* < 0.001) at 60 min after FSK perfusion and lasted for at least 100 min. However, propofol (50 μM) pretreatment for 20 min significantly inhibited the FSK induced increases of the fEPSP slope (108 ± 8% (*n* = 6) of baseline vs. 139 ± 8% (*n* = 6) in FSK alone group, *p* < 0.05) (Figure 1). These results suggest that propofol is likely to influence the cAMP pathway during the LTP induction in the hippocampal CA1 region.

### cAMP level decreased by propofol was due to the increase of both PDE activity and protein level

**Figure 2:**
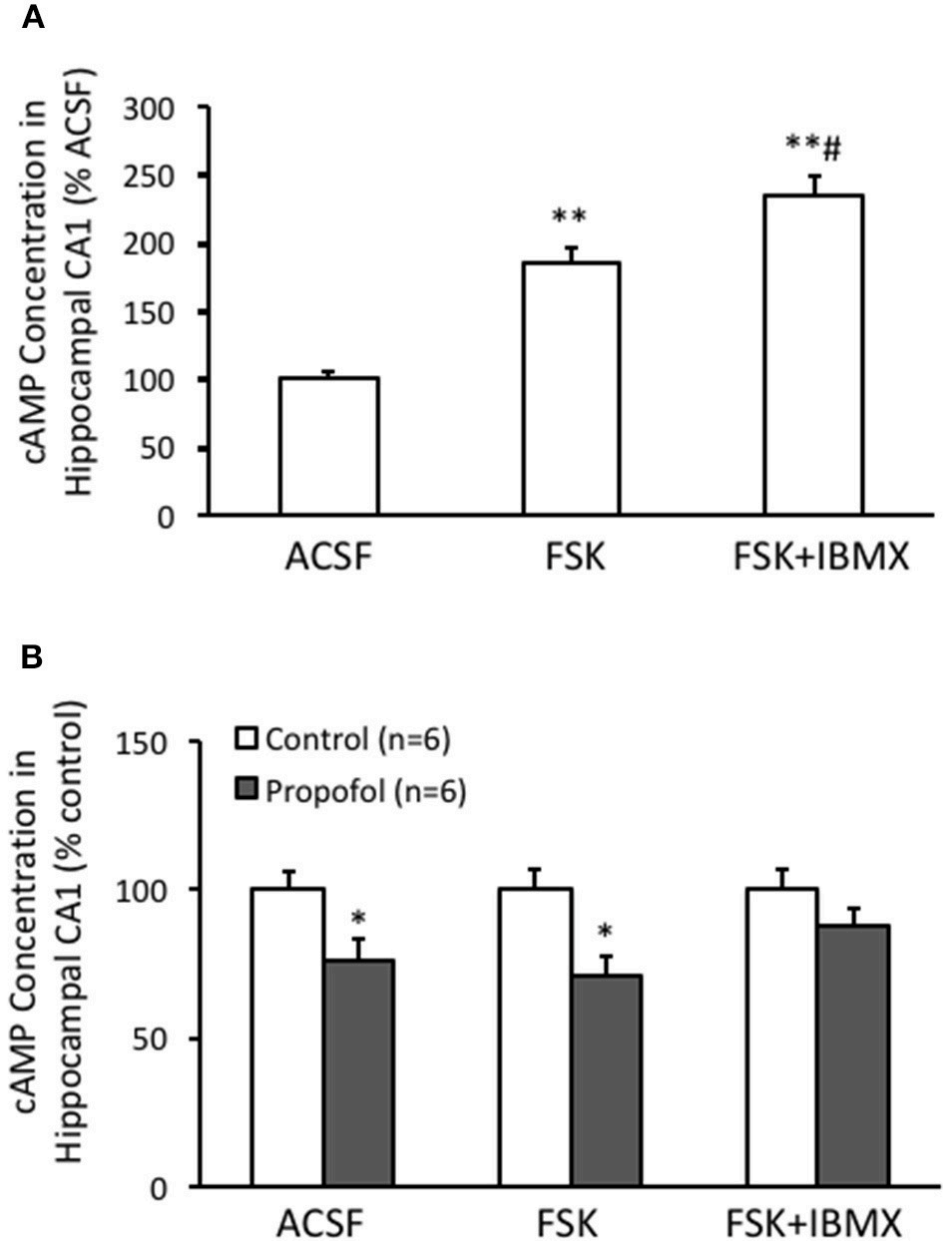
PDE inhibitor IBMX rescues propofol caused decrease of cAMP level in the hippocampal CA1. **(A)** Bar histograms showing the relative cAMP level, normalized to level in ACSF as 100%, of hippocampal CA1 cells incubated with either ACSF, FSK or FSK+IBMX (*n* = 6 in each group, respectively) for 15 min. ^**^p < 0.01 compared with ACSF, ^#^*p* < 0.05 compared with FSK group. **(B)** Bar histogram showing propofol incubation significantly reduced the cAMP concentration in the hippocampal CA1, and co-incubation with FSK and IBMX together but not FSK alone prevented the reduction induced by propofol. ^*^p < 0.05 compared with its relative control group, *n* = 6 in each group.

Propofol decreases the cAMP level and inhibits cAMP signaling, but its underlying mechanism is little to be known. Cell cAMP level is positively regulated by adenylyl cyclases (ACs) and negatively affected by cyclic nucleotide phosphodiesterases (PDEs). In this aspect, we performed experiments to test whether stimulating ACs by FSK, or inhibition of PDEs by IBMX, would affect the cAMP level in either normal or propofol treatment conditions. Our experimental results showed that FSK treatment significantly increased the cAMP level to 184 ± 12% (*n* = 6) of ACSF control level (*P* < 0.01 compared with ACSF group) in hippocampal CA1 cells, and there was a further increase of cAMP level to 233 ± 15% (*n* = 6) of ACSF control level while IBMX was added on top of the FSK treatment (*P* < 0.01 compared with ACSF control and *P* < 0.05 compared with FSK alone group) (Figure 2A). This result suggested that both FSK, by increasing the cAMP synthesize, and IBMX, by enhancing cAMP degradation, could influence the intracellular cAMP level in hippocampus. Next, we tested whether FSK or IBMX could reverse propofol induced decrease of cAMP in hippocampus. Similar as previous reported, propofol alone significantly decreased cAMP concentration in the hippocampal CA1 region (75 ± 7% of control, *n* = 6, *p* < 0.05) (Figure 2B). In the slices of FSK treatment, propofol could also decrease cAMP concentration (71 ± 7% of control *n* = 6, *p* < 0.05, Figure 2B). In contrast, when co-treatment FSK together with IBMX, the cAMP concentration in the presence of propofol was no longer significantly reduced (87 ± 6% of ACSF control, *p* = 0.19) (Figure 2B). These results indicated that blockade of cAMP synthesize with FSK could not, but suppressing cAMP degradation by inhibiting PDE activity, could reverse propofol induced cAMP concentration deduction.

**Figure 3:**
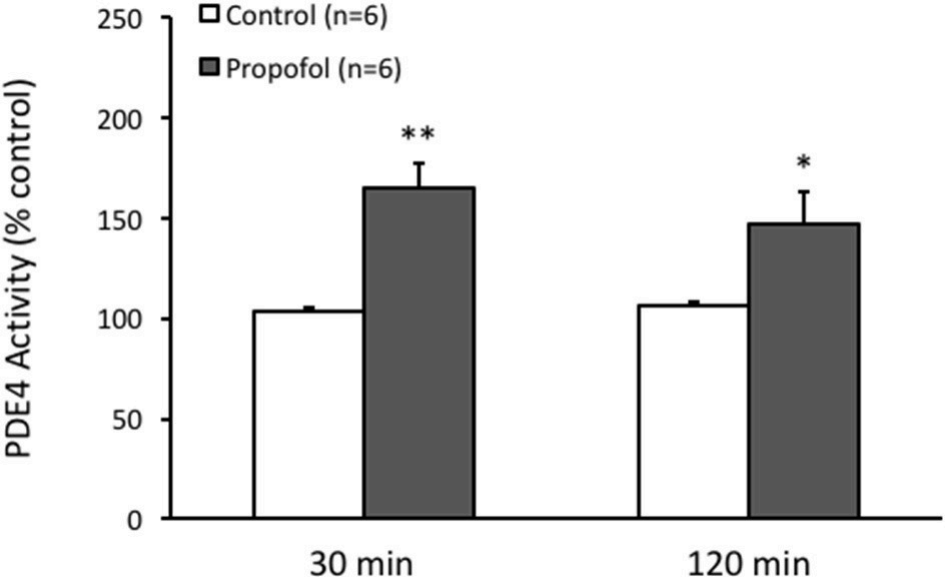
Propofol increases the PDE4 activity in the hippocampal CA1 of rats. Bar histograms showing the ELISA measured PDE4 activity in the hippocampal CA1 area significantly increased 30 min and 120 min after propofol injection (50 mg/kg, i.p.) in rats. ^*^p < 0.05, ^**^p < 0.01 compared with vehicle control group by Student's *t*-test.

Since PDEs are enzymes that provide the sole route for cAMP degradation in cells and PDE4 has a major role in the brain cells. Therefore, we speculated that the reduction of the cAMP level in the hippocampal CA1 region by propofol was related to PDE, particularly PDE4, pathway. Indeed by measuring the PDE4 activity in the hippocampus using Enzyme Immunoassay, we observed that PDE4 activity in the hippocampus was significantly enhanced in the presence of propofol. The PDE4 activity in the hippocampus was increased to 164 ± 13% (*n* = 6, *P* < 0.01) and 147 ± 15% (*n* = 6, *P* < 0.05) of ACSF control level at either 30 or 120 min after propofol administration, respectively (Figure 3).

**Figure 4:**
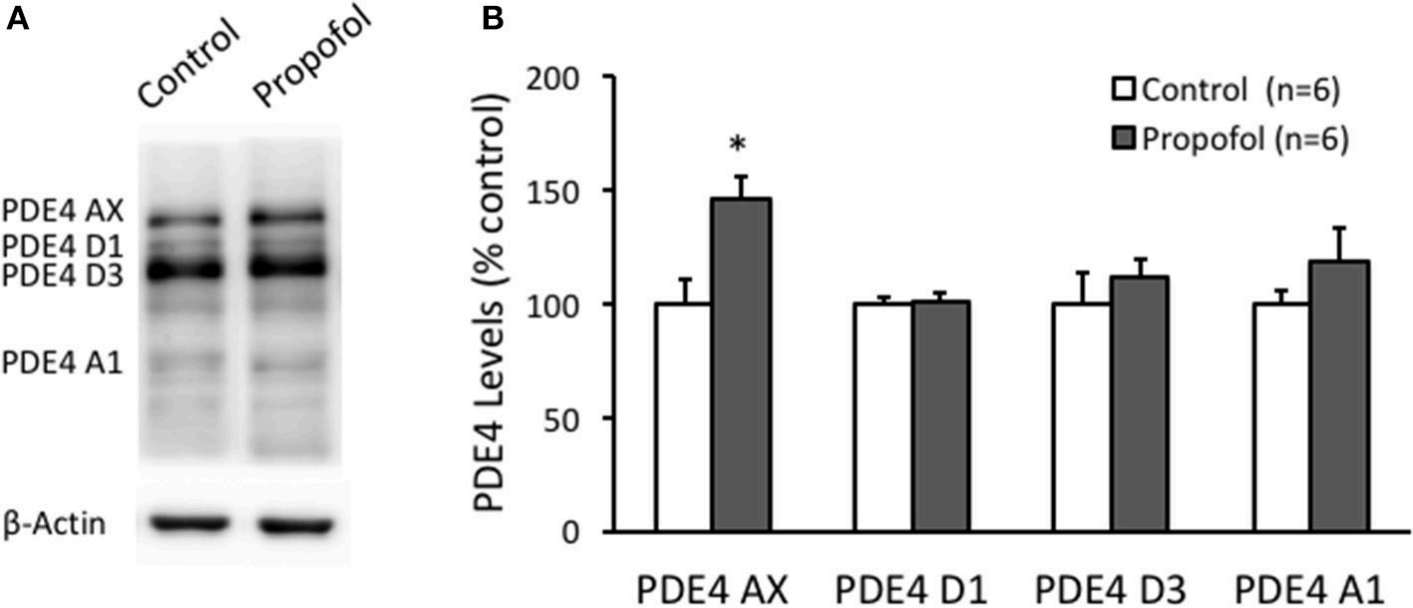
Propofol increases the PDE4AX protein levels in the hippocampal CA1 of rats. **(A)** Representative WB bands showing PDE4 isoforms, PDE4AX, PDE4D1, PDE4D3, and PDE4A1 are detected from samples of rat hippocampus. **(B)** Bar histograms showing the PDE4 AX protein levels in hippocampus significantly increased 120 min after propofol injection (50 mg/kg, i.p.) in rats. The other isoforms in hippocampus had no significant changes. Data were shown as mean ± SEM. ^*^p < 0.05, compared with vehicle control group by Student's *t*-test.

Next, we investigated the PDE4 isoforms protein expression in the hippocampal CA1 region by western blot. PDE4 AX, D1, D3, and A1 were detected by using a pan-PDE4 antibody. PDE4 AX level in the hippocampal CA1 region was found significantly increased in propofol group (146 ± 9% of control, *n* = 6, *P* = 0.01), but not all the other subtypes (Figure 4). These results suggested that propofol suppressing the cAMP level in the hippocampus is likely due to the increase of both the activity and the protein level of the PDE4, and in turn to reduce the cAMP degradation rate.

### Rolipram reversed propofol induced LTP inhibition in the hippocampal CA1 region

**Figure 5:**
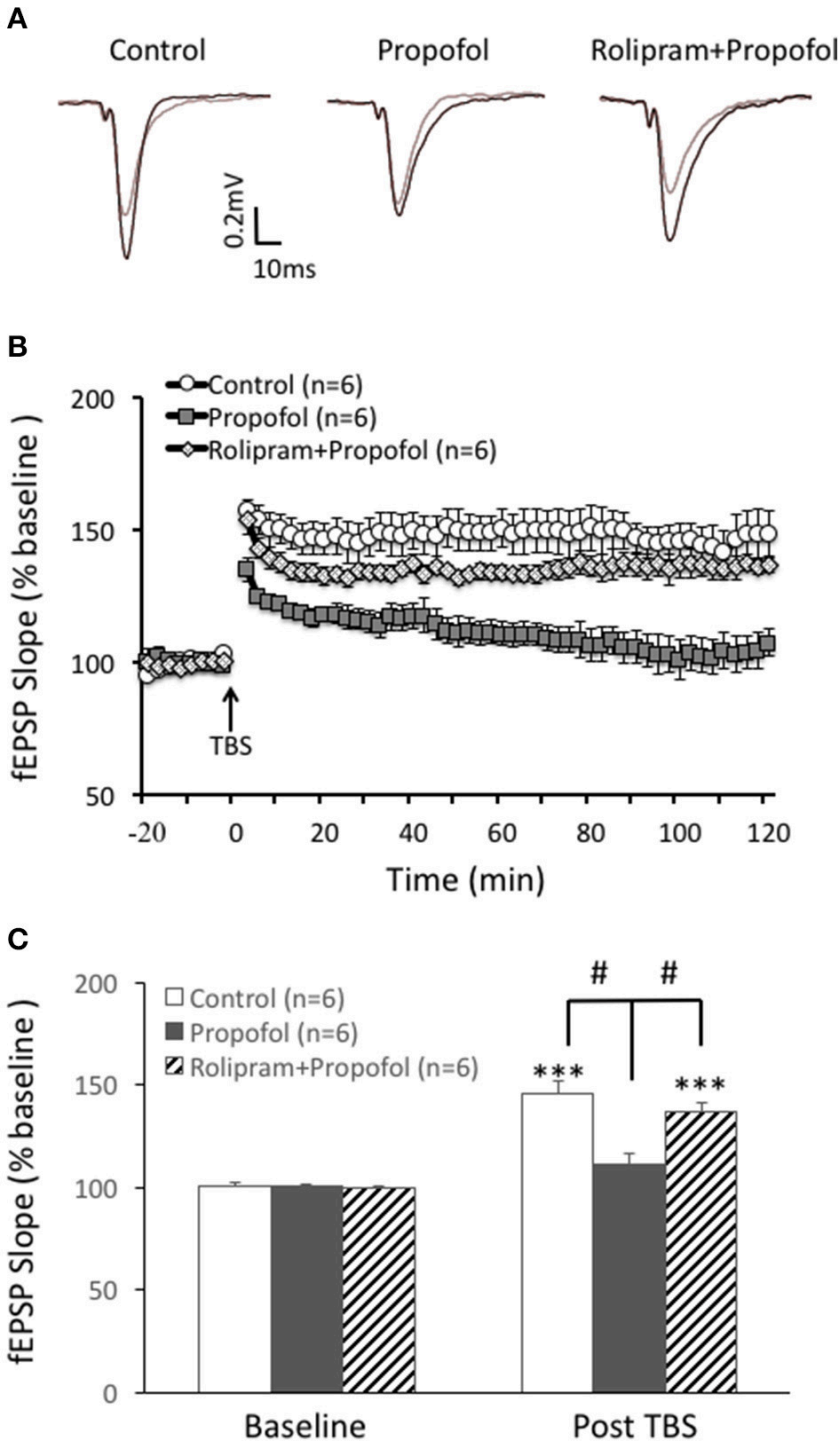
Rolipram rescues propofol induced LTP inhibition in the hippocampal CA1. **(A)** Raw traces showing of the recording of the evoked field potential 5min before (gray line) and 80min after the LTP induction (black line). **(B)** Theta burst stimulation (TBS) induced long term potentiation (open circle) in the rats' hippocampal CA1 area. Propofol (30 μM) perfused for 20 min before TBS significantly inhibited the LTP induction (black square) and rolipram co-treatment rescued propofol caused inhibition of the CA1 LTP (doted diamond square). **(C)** Bar histograms showing the group data (baseline and post-TBS) of the propofol effect on TBS induced LTP taken from 80 to 90 min after TBS, and the rescue effect of rolipram on the propofol inhibition of hippocampal CA1 LTP. ^***^p < 0.001 compared with baseline by student *t*-test, and ^#^*p* < 0.05 compared with the propofol group by one way ANOVA with *post-hoc* tests.

Based on the above result that propofol enhanced the PDE 4 activity in the hippocampal CA1 region, we further investigated whether increased PDE4 activity was responsible for propofol induced inhibition of LTP in the hippocampal CA1 region. Similar to our previous findings, propofol 30 μM) significantly inhibited TBS induced hippocampal CA1 LTP (110 ± 6% of the baseline in propofol group vs. 146 ± 6% of the baseline in control group at 60 min after TBS, *n* = 6 in each group, *p* < 0.01; Figure 5). However, rolipram perfused together with the propofol, significantly reversed the suppression of LTP by propofol in the hippocampal CA1 slices. Rolipram increased TBS induced hippocampal CA1 LTP from 110 ± 6% (*n* = 6) of the baseline in the propofol group to 133 ± 5% (*n* = 6) of the baseline in the propofol plus rolipram group (*p* < 0.01; Figure 5). This result showed that PDE4 inhibitor could rescue propofol induced inhibition of LTP in the hippocampal CA1 region, which suggested that PDE4 pathway impairment was responsible for propofol induced LTP inhibition.

### Rolipram rescued contextual fear memory impairment caused by propofol in rats

Contextual fear conditioning was employed to exam hippocampal dependent memory formation in this experiment. During the memory tasks, freezing behavior was used as an indicator of rats' recognition of a potentially aversive stimulus, and thus an indicator of memory function. Thirty-six male rats (10–12 weeks) were randomly divided into four groups. Rats received propofol treatment (25 mg/kg, i.p.), rolipram treatment (1 mg/kg, i.p.), or vehicle (2% DMSO in 0.9% saline) respectively at 30 min before undergoing behavior test.

**Figure 6:**
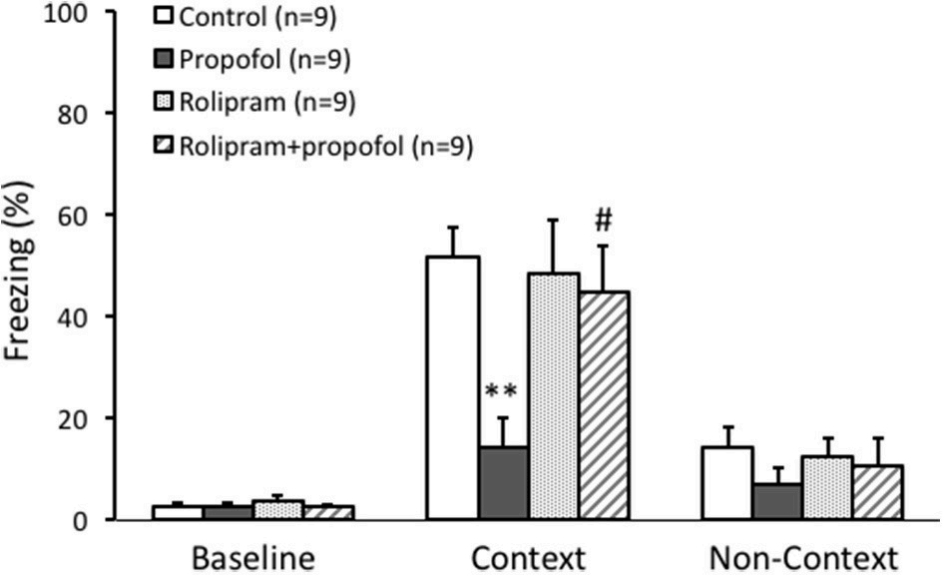
Rolipram rescued contextual fear memory impairment caused by propofol in rats. Fear conditioning tested the context specific memory of rats. The freezing percentage before training was similar between four groups. During the context test, the percentage of freezing in propofol group was significantly lower than it in control group (*p* < 0.01). Combined rolipram injection with propofol rescued the percentage of freezing decrease induced by propofol (*p* < 0.05). ^**^p < 0.01 compared with Control group, ^#^*p* < 0.05 compared with propofol group by one way ANOVA with *post-hoc* test.

The baseline of freezing behavior indicated by the freezing percentage before training on day 1 was similar among the four groups (*p* > 0.05, Figure 6). During the task tests on day 2 in the contexture test, the percentage of freezing time in propofol group was significantly lower than that of control group (14 ± 6% vs. 51 ± 6%, *n* = 9, *p* < 0.01, Figure 6). However, combined injection of rolipram with propofol rescued the decreased freezing percentage induced by propofol (44 ± 9%, *n* = 9, *p* < 0.05, Figure 6). There was no significant difference of freezing behavior among the four groups on the non-contextual tests on day 2 (*p* > 0.05, Figure 6). The ability of rolipram to rescue propofol induced impairment in hippocampus dependent memory formation demonstrated that propofol increasing of PDE4 activities and inhibiting cAMP signaling in the hippocampal CA1 region is likely, at least in part, responsible for the memory impairment effects of propofol *in vivo*.

## Discussion

In this study, we demonstrated that propofol inhibited cAMP signaling dependent LTP in the CA1 region of rats' hippocampus through suppressing cAMP levels as a result of increased PDE4 activity and PDE4AX protein level. And treatment of PDE4 inhibitor rolipram rescued propofol induced CA1 LTP inhibition and memory formation impairment in rats. These results showed that the suppression of cAMP signaling by increasing PDE4 activity and protein expression in the hippocampal CA1 region played a major role in the propofol induced amnesia.

Animal studies have shown that cAMP-CREB pathway in the hippocampus is crucial for the LTP and memory formation (Suzuki et al., 2011; Kida, 2012; Kida and Serita, 2014). It had been reported that propofol inhibited the phosphorylation of the CREB, a transcriptional activator, in the hippocampus and down regulated the BDNF and Arc protein expression (Zhang et al., 2013). In the present study, we obtained several sets of data about the role of the key molecular cAMP in the propofol induced LTP inhibition and memory impairment. First, consistent with the results that propofol inhibited cAMP dependent LTP in the hippocampal CA1 region (Nagashima et al., 2005; Li et al., 2014), propofol inhibited the FSK induced LTP in the hippocampal CA1 region. It further endorsed the assumption that propofol inhibited cAMP signaling induced CA1 LTP. Second, propofol reduced the cAMP level in the rats' hippocampus, which provided direct evidence that propofol could inhibit cAMP signaling in the rats' hippocampus. Third, PDE4 inhibitor could rescue propofol induced cAMP dependent LTP inhibition in the hippocampal CA1 region. Taken together, these results indicated that propofol inhibited cAMP signaling in the hippocampal CA1 region was involved in its effects on LTP formation.

Many factors could modulate cAMP levels in cells mainly through two series of enzymes ACs and PDEs. For example, numerous agents such as FSK and pertussis toxin increased the cAMP levels in brain cells by activating AC directly or indirectly (Ahuja et al., 2004; Vecsey et al., 2009). PDE activity enhanced or inhibited also affects the cAMP levels in neurons (Kleppisch, 2009; Xu et al., 2011). In this study, the reduced cAMP levels by propofol could be partly reversed by a broad-spectrum PDEs inhibitor IBMX, but not AC activator FSK, which suggested that the effect of propofol on reduced cAMP levels was due to PDE inhibition. The cAMP-specific PDE4 in the brain played an important role in regulating cAMP signaling (Houslay and Adams, 2003; Kleppisch, 2009). Then we found that propofol could increase the PDE 4 activity in the hippocampus in rats. Additionally, the PDE 4 selective inhibitor rolipram could rescue cAMP dependent LTP inhibition and memory formation by propofol treatment. These results may be taken to endorse the hypothesis that propofol reduced the cAMP levels in the hippocampal CA1 region and inhibited the LTP and memory formation are at least in part by increased PDE4 activity.

Fear conditioning was employed in this study to assess the ability of memory formation in rats. The test paradigm included contextual fear conditioning which required normal hippocampal function (Rudy et al., 2004; Alvarez et al., 2008) and cued fear conditioning which relied heavily on the amygdala (Johansen et al., 2011; Bergstrom et al., 2013). During the contextual fear conditioning test, we found that both propofol and rolipram alone had little effects on learning course. However, propofol (25 mg/kg, i.p.) significantly reduced the freezing time during the task test, indicated that even subanesthesia dose propofol impaired contextual memory formation. Rolipram alone couldn't enhance the memory of normal animals in our study, which agreed with the reported study by Barad and his colleagues (Barad et al., 1998). It may be related to that rolipram had no significant effect on basal cAMP concentration. However, similar to reported results in water maze experiments (Zhang et al., 2013), rolipram treatment before propofol administration rescued the contextual memory function impaired by propofol in rats. There are two factors which may contribute to this effect *in vivo*. On one hand, propofol induced cAMP reduce can be reversed by IBMX. It could be speculated that rolipram also could in part reversed the cAMP reduction. On the other, many reports had shown that rolipram could facilitate LTP induction and transform early LTP to late LTP. In this study, it rescued propofol induced LTP inhibition in the hippocampal slice (Barad et al., 1998; Navakkode et al., 2004).

In addition, we found the similar results in the cued fear conditioning experiment. Propofol also impaired the memory formation and reduced by rolipram in rats. Because amygdala, but not hippocampus, plays more of an exclusive role in cue fear conditioning (Johansen et al., 2011; Bergstrom et al., 2013), these results suggested that propofol and rolipram also worked on other areas in the brain such as amygdala. Indeed, it had reported that amygdala was involved in the propofol-induced amnesia (Ren et al., 2008, 2015). And administration of rolipram into the amygdala at a specific time interval after training enhances memory persistence for novel object recognition in rats (Werenicz et al., 2012). All these showed that it should be paid attention to some other memory related areas in the brain that may also play an important role in the effect of propofol and rolipram.

There are some limitations in the study. First, although PDE4 seems to be the most promising target in the brain, there are many other PDEs such as PDE 7 and PDE 8 could play a role in propofol induced cAMP reduction (Heckman et al., 2015). Second, we investigated four PDE4 isoforms by western blot but we did not know whether other isoforms changed. In addition, rolipram was intraperitoneally injected but not locally in behavior tests. As PDE4 was ubiquitously expressed in the brain regions such as amygdala, prefrontal cortex, and basal forebrain, might be also contributed to the effects of rolipram. These possibilities should be included for consideration when interpreting our results.

This study and our previous findings provided further information about the cellular and molecular mechanisms of propofol produced amnesia. It revealed the major role of cAMP signaling and PDE4 activity in the hippocampal CA1 region on propofol induced changes of hippocampal function as well as memory formation. Except the theoretical significance, it also showed practical values in the clinical experiences. Although a great number of patients underwent surgery benefited from propofol-produced amnesia, it would be an adverse effect if some patients underwent short examinations such as gastrointestinal endoscopy recovers slowly. It was reported that memory remain depressed for several hours in these patients after cessation of propofol administration (Korttila et al., 1992). What we found in this study suggested that modification of PDE4 activity and cAMP signaling should have a potential effect to promote memory recovery after propofol administration.

In the current study, we demonstrated that propofol could increase the activity of PDE4 and also the protein level of PDE4AX, which resulted in the decrease of cAMP level in the hippocampal CA1 region. The decrease of cAMP level in the hippocampal CA1 may contribute to the propofol induced amnesia. This was supported by the results that rolipram, a selective PDE4 inhibitor, rescued propofol induced LTP inhibition in the hippocampal CA1 region and amnesia in rats. In summary, our current results in this paper discovered that PDE4 in the hippocampal CA1 region plays a key role in the propofol induced amnesia in rats.

## Author contributions

WL, LY, YW, and HX conceived the idea and designed the experiments. WL, LY, XY, LC, LW, and YL performed the experiments and interpreted the experimental results. WL, LY, YW, and HX participated in the study design and data analysis. WL, LY, and YW wrote the manuscript. All authors read and approved the current version of the manuscript.

### Conflict of interest statement

The authors declare that the research was conducted in the absence of any commercial or financial relationships that could be construed as a potential conflict of interest.

## References

[B1] Ahuja N. Kumar P. Bhatnagar R. (2004). The adenylate cyclase toxins. Crit. Rev. Microbiol. 30, 187–196. 10.1080/10408410490468795 15490970

[B2] Alvarez R. P. Biggs A. Chen G. Pine D. S. Grillon C. (2008). Contextual fear conditioning in humans: cortical-hippocampal and amygdala contributions. J. Neurosci. 28, 6211–6219. 10.1523/JNEUROSCI.1246-08.2008 18550763 PMC2475649

[B3] Barad M. Bourtchouladze R. Winder D. G. Golan H. Kandel E. (1998). Rolipram, a type IV-specific phosphodiesterase inhibitor, facilitates the establishment of long-lasting long-term potentiation and improves memory. Proc. Natl. Acad. Sci. U.S.A. 95, 15020–15025. 10.1073/pnas.95.25.15020 9844008 PMC24568

[B4] Bergstrom H. C. McDonald C. G. Dey S. Tang H. Selwyn R. G. Johnson L. R. (2013). The structure of Pavlovian fear conditioning in the amygdala. Brain Struct. Funct. 218, 1569–1589. 10.1007/s00429-012-0478-2 23179863

[B5] Costa A. C. Grybko M. J. (2005). Deficits in hippocampal CA1 LTP induced by TBS but not HFS in the Ts65Dn mouse: a model of Down syndrome. Neurosci. Lett. 382, 317–322. 10.1016/j.neulet.2005.03.031 15925111

[B6] Curzon P. Rustay N. R. Browman K. E. (2009). Chapter 2: Cued and contextual fear conditioning for rodents, in Methods of Behavior Analysis in Neuroscience, 2nd Edn, ed Buccafusco J. J. (Boca Raton, FL: CRC Press/Taylor & Francis). 21204331

[B7] Eichenbaum H. (1996). Learning from LTP: a comment on recent attempts to identify cellular and molecular mechanisms of memory. Learn. Mem. 3, 61–73. 10.1101/lm.3.2-3.61 10456077

[B8] Heckman P. R. Blokland A. Ramaekers J. Prickaerts J. (2015). PDE and cognitive processing: beyond the memory domain. Neurobiol. Learn. Mem. 119, 108–122. 10.1016/j.nlm.2014.10.011 25464010

[B9] Houslay M. D. Adams D. R. (2003). PDE4 cAMP phosphodiesterases: modular enzymes that orchestrate signalling cross-talk, desensitization and compartmentalization. Biochem. J. 370, 1–18. 10.1042/bj20021698 12444918 PMC1223165

[B10] Johansen J. P. Cain C. K. Ostroff L. E. LeDoux J. E. (2011). Molecular mechanisms of fear learning and memory. Cell 147, 509–524. 10.1016/j.cell.2011.10.009 22036561 PMC3215943

[B11] Kandel E. R. (2012). The molecular biology of memory: cAMP, PKA, CRE, CREB-1, CREB-2, and CPEB. Mol. Brain 5:14. 10.1186/1756-6606-5-14 22583753 PMC3514210

[B12] Kida S. (2012). A functional role for CREB as a positive regulator of memory formation and LTP. Exp. Neurobiol. 21, 136–140. 10.5607/en.2012.21.4.136 23319873 PMC3538177

[B13] Kida S. Serita T. (2014). Functional roles of CREB as a positive regulator in the formation and enhancement of memory. Brain. Res. Bull. 105, 17–24. 10.1016/j.brainresbull.2014.1004.1011 24811207

[B14] Kleppisch T. (2009). Phosphodiesterases in the central nervous system. Handb. Exp. Pharmacol. 191, 71–92. 10.1007/978-3-540-68964-5_5 19089326

[B15] Korttila K. Nuotto E. J. Lichtor J. L. Ostman P. L. Apfelbaum J. Rupani G. (1992). Clinical recovery and psychomotor function after brief anesthesia with propofol or thiopental. Anesthesiology 76, 676–681. 10.1097/00000542-199205000-00003 1575333

[B16] Li W. W. Cheng L. Z. Zou Z. Tian M. L. Zhang H. Raya A. D.. (2014). (R)-alpha-methylhistamine suppresses inhibitory neurotransmission in hippocampal CA1 pyramidal neurons counteracting propofol-induced amnesia in rats. CNS Neurosci. Ther. 20, 851–859. 10.1111/cns.12294 24948006 PMC6493288

[B17] Miller S. Mayford M. (1999). Cellular and molecular mechanisms of memory: the LTP connection. Curr. Opin. Genet. Dev. 9, 333–337. 10.1016/S0959-437X(99)80050-1 10377283

[B18] Moscovitch M. Cabeza R. Winocur G. Nadel L. (2016). Episodic memory and beyond: the hippocampus and neocortex in transformation. Annu. Rev. Psychol. 67, 105–134. 10.1146/annurev-psych-113011-143733 26726963 PMC5060006

[B19] Nagashima K. Zorumski C. F. Izumi Y. (2005). Propofol inhibits long-term potentiation but not long-term depression in rat hippocampal slices. Anesthesiology 103, 318–326. 10.1097/00000542-200508000-00015 16052114

[B20] Navakkode S. Sajikumar S. Frey J. U. (2004). The type IV-specific phosphodiesterase inhibitor rolipram and its effect on hippocampal long-term potentiation and synaptic tagging. J. Neurosci. 24, 7740–7744. 10.1523/JNEUROSCI.1796-04.2004 15342741 PMC6729613

[B21] Nguyen P. V. Kandel E. R. (1997). Brief theta-burst stimulation induces a transcription-dependent late phase of LTP requiring cAMP in area CA1 of the mouse hippocampus. Learn. Mem. 4, 230–243. 10.1101/lm.4.2.230 10456066

[B22] Nguyen P. V. Woo N. H. (2003). Regulation of hippocampal synaptic plasticity by cyclic AMP-dependent protein kinases. Prog. Neurobiol. 71, 401–437. 10.1016/j.pneurobio.2003.12.003 15013227

[B23] Pang R. Quartermain D. Rosman E. Turndorf H. (1993). Effect of propofol on memory in mice. Pharmacol. Biochem. Behav. 44, 145–151. 10.1016/0091-3057(93)90292-2 8430117

[B24] Ren Y. Wang J. Xu P. B. Xu Y. J. Miao C. H. (2015). Systemic or intra-amygdala infusion of an endocannabinoid CB1 receptor antagonist AM251 blocked propofol-induced anterograde amnesia. Neurosci. Lett. 584, 287–291. 10.1016/j.neulet.2014.1011.1001 25445359

[B25] Ren Y. Zhang F. J. Xue Q. S. Zhao X. Yu B. W. (2008). Bilateral inhibition of gamma-aminobutyric acid type A receptor function within the basolateral amygdala blocked propofol-induced amnesia and activity-regulated cytoskeletal protein expression inhibition in the hippocampus. Anesthesiology 109, 775–781. 10.1097/ALN.0b013e31818a37c4 18946287

[B26] Rudy J. W. Huff N. C. Matus-Amat P. (2004). Understanding contextual fear conditioning: insights from a two-process model. Neurosci. Biobehav. Rev. 28, 675–685. 10.1016/j.neubiorev.2004.09.004 15555677

[B27] Staubli U. Scafidi J. Chun D. (1999). GABAB receptor antagonism: facilitatory effects on memory parallel those on LTP induced by TBS but not HFS. J. Neurosci. 19, 4609–4615. 10.1523/JNEUROSCI.19-11-04609.1999 10341258 PMC6782628

[B28] Suzuki A. Fukushima H. Mukawa T. Toyoda H. Wu L. J. Zhao M. G.. (2011). Upregulation of CREB-mediated transcription enhances both short- and long-term memory. J. Neurosci. 31, 8786–8802. 10.1523/JNEUROSCI.3257-10.2011 21677163 PMC6622960

[B29] Takamatsu I. Sekiguchi M. Wada K. Sato T. Ozaki M. (2005). Propofol-mediated impairment of CA1 long-term potentiation in mouse hippocampal slices. Neurosci. Lett. 389, 129–132. 10.1016/j.neulet.2005.07.043 16112456

[B30] Vecsey C. G. Baillie G. S. Jaganath D. Havekes R. Daniels A. Wimmer M.. (2009). Sleep deprivation impairs cAMP signalling in the hippocampus. Nature 461, 1122–1125. 10.1038/nature08488 19847264 PMC2783639

[B31] Veselis R. A. Reinsel R. A. Feshchenko V. A. Johnson R. Jr. (2004). Information loss over time defines the memory defect of propofol: a comparative response with thiopental and dexmedetomidine. Anesthesiology 101, 831–841. 10.1097/00000542-200410000-00006 15448514 PMC1404599

[B32] Wang H. Ferguson G. D. Pineda V. V. Cundiff P. E. Storm D. R. (2004). Overexpression of type-1 adenylyl cyclase in mouse forebrain enhances recognition memory and LTP. Nat. Neurosci. 7, 635–642. 10.1038/nn1248 15133516

[B33] Werenicz A. Christoff R. R. Blank M. Jobim P. F. Pedroso T. R. Reolon G. K.. (2012). Administration of the phosphodiesterase type 4 inhibitor rolipram into the amygdala at a specific time interval after learning increases recognition memory persistence. Learn. Mem. 19, 495–498. 10.1101/lm.026997.112 22993171

[B34] Wixted J. T. Squire L. R. Jang Y. Papesh M. H. Goldinger S. D. Kuhn J. R.. (2014). Sparse and distributed coding of episodic memory in neurons of the human hippocampus. Proc. Natl. Acad. Sci. U.S.A. 111, 9621–9626. 10.1073/pnas.1408365111 24979802 PMC4084456

[B35] Wong S. T. Athos J. Figueroa X. A. Pineda V. V. Schaefer M. L. Chavkin C. C.. (1999). Calcium-stimulated adenylyl cyclase activity is critical for hippocampus-dependent long-term memory and late phase LTP. Neuron 23, 787–798. 10.1016/S0896-6273(01)80036-2 10482244

[B36] Xu Y. Zhang H. T. O'Donnell J. M. (2011). Phosphodiesterases in the central nervous system: implications in mood and cognitive disorders. Handb. Exp. Pharmacol. 204, 447–485. 10.1007/978-3-642-17969-3_19 21695652

[B37] Yu T. P. McKinney S. Lester H. A. Davidson N. (2001). Gamma-aminobutyric acid type A receptors modulate cAMP-mediated long-term potentiation and long-term depression at monosynaptic CA3-CA1 synapses. Proc. Natl. Acad. Sci. U.S.A. 98, 5264–5269. 10.1073/pnas.091093998 11296264 PMC33198

[B38] Zhang H. Zhang S. B. Zhang Q. Q. Liu M. He X. Y. Zou Z.. (2013). Rescue of cAMP response element-binding protein signaling reversed spatial memory retention impairments induced by subanesthetic dose of propofol. CNS Neurosci. Ther. 19, 484–493. 10.1111/cns.12088 23534694 PMC6493409

